# A Descriptive Longitudinal Study of Changes in Vape Shop Characteristics and Store Policies in Anticipation of the 2016 FDA Regulations of Tobacco Products, Including E-Cigarettes

**DOI:** 10.3390/ijerph15020313

**Published:** 2018-02-11

**Authors:** Sheila Yu, Patricia Escobedo, Robert Garcia, Tess Boley Cruz, Jennifer B. Unger, Lourdes Baezconde-Garbanati, Leah Meza, Steve Sussman

**Affiliations:** 1 Department of Preventive Medicine, University of Southern California, 2001 N Soto St., Los Angeles, CA 90032, USA; pescobed@usc.edu (P.E.); garc617@usc.edu (R.G.); tesscruz@usc.edu (T.B.C.); unger@usc.edu (J.B.U.); baezcond@usc.edu (L.B.-G.); leahmedi@usc.edu (L.M.); ssussma@usc.edu (S.S.); 2 Department of Psychology, University of Southern California, 3620 South McClintock Ave, Los Angeles, CA 90089, USA; 3 School of Social Work, University of Southern California, Montgomery Ross Fisher Building, Los Angeles, CA 90089, USA

**Keywords:** electronic cigarettes, vape shops, FDA, Deeming Rule, longitudinal, public health

## Abstract

After proposing the “Deeming Rule” in 2014, the U.S. Food and Drug Administration (FDA) began regulating the manufacturing, marketing, and sales of electronic cigarette (e-cigarette) products as tobacco products in 2016. The current study conducted vape shop store observations and surveyed Los Angeles–area shop employees (assessing their beliefs, awareness, and perceptions of e-cigarettes and related FDA regulations) at two time points one year apart to better understand what vape shop retailers would do given FDA’s soon-to-be-enacted Deeming Rule. The study also compared retailer beliefs/awareness/actions and store characteristics immediately after the Deeming Rule proposal versus a year after the Rule had been proposed, right before its enactment. Two data collection waves occurred before the Deeming Rule enactment, with Year 1 surveying 77 shops (2014) and Year 2 surveying 61 shops (2015–2016). Between the data collection points, 16 shops had closed. Among the shops that were open at both time points, the majority (95% in Year 1; 74% in Year 2) were aware of some FDA regulations or other policies applying to vape shops. However, overall awareness of FDA regulations and state/local policies governing e-cigarettes significantly decreased from Year 1 to Year 2. At both time points, all shops offered customers free puffs of nicotine-containing e-liquids (prohibited by the then upcoming Deeming Rule). Perceptions of e-cigarette safety also significantly decreased between the years. Exploring vape shop retailer perceptions and store policies (i.e., free puffs/samples displays, perceptions of e-cigarette safety, etc.) over time will help the FDA assess the needs of the vape shop community and develop more effective retailer education campaigns and materials targeted to increase compliance with the newly enacted regulations.

## 1. Introduction

Electronic cigarettes (e-cigarettes) are popular among current, former, and non-cigarette-smokers, and particularly appealing to youth and young adults [1,2,3,4,5]. The overall growth in popularity of e-cigarettes has resulted in a recent surge of new vape shops throughout the United States and around the world [1,2,3,4]. The extent to which vape shops contribute to the initiation of e-cigarette use by current, former, and non-cigarette-smokers, youth, and other vulnerable populations remains unknown. Some research suggests that vape shop retailers may play a part in promoting lifestyle changes among customers who smoke, i.e., promoting e-cigarette use while ceasing combustible cigarette use, although retailers may also offer customers unsubstantiated tips and claims related to health outcomes [3,6]. Therefore, the recent proliferation in vape shops indicates the need for guidance on how to increase vape shop retailer awareness of and compliance with Food and Drug Administration (FDA) regulations and with state/local rules related to e-cigarette products and shops. Observing and comparing what vape shops do immediately after a proposed rule until close to its enactment and examining changes in vape shop activities over a period of time could help guide local and federal activity to support the vape shops.

It is also critical to examine differences in vape shop activities based on ethnic location, which could uncover ethnic location-specific characteristics that FDA needs to consider when communicating tobacco regulatory policies and regulations with retailers. This study looks at differences in ethnic locations (communities with a relatively high percentage of Korean, Hispanic/Latino, African American, or non-Hispanic White residents) since these particular population groups are known to be vulnerable to tobacco use [7,8,9]. Although Hispanic/Latino adults have lower prevalence of cigarette smoking and other tobacco use than other racial/ethnic groups in general, tobacco use continues to be a health risk among this group, and use of e-cigarettes and hookah has increased among younger Hispanic/Latino tobacco users living in the U.S. [10]. 

The manufacturing, marketing, and sales of e-cigarette products in the U.S. were not under the jurisdiction of federal regulations until May 2016 when the FDA published its finalized Deeming Rule, granting the FDA federal regulatory authority over e-cigarettes, cigars, and hookah tobacco products equivalent to the rules in place for cigarettes and smokeless tobacco [5]. The Deeming Rule, effective as of 8 August 2016, prohibits vape shop retailers from selling e-cigarettes to individuals younger than 18 years of age and requires photo identification to verify age of all customers under 27 years old. Additionally, the new regulation prohibits all retailers from selling e-cigarettes or related vape products in vending machines and giving away free e-cigarette samples or samples of related vape products, including any part and/or component of those products [11]. 

The FDA now classifies a vape shop as both a retailer if it sells e-cigarette liquids and equipment, and as a tobacco product manufacturer if it mixes its own e-liquids (juices for e-cigarettes and vape products), makes or modifies vaporizers, or combines loose tobacco with the e-cigarette or other vape products [11]. Manufacturing entails additional rules and restrictions for the vape shop. Shops that engage in these latter activities will need to register as a tobacco product manufacturer (which involves additional licenses and fees), disclose lists of all products and their ingredients, and be subject to pre-market review and authorization of each product by the FDA [5].

Recently, the Deeming Rule was challenged by Nicopure Labs, LLC, an e-cigarette and vaping-related product manufacturer, claiming that the rule “exceeded the agency’s statutory authority under the Food, Drug, and Cosmetic Act, 21 U.S.C. § 301, et seq. (“FDCA”), as amended by the Family Smoking Prevention and Tobacco Control Act, Pub. L. No. 111-31, 123 Stat. 1777 (2009) (“Tobacco Control Act” or “TCA”) [12]”. Ten pro-vaping coalitions (collectively known as the RSF, “Right to be Smoke Free” Coalition) also independently challenged the rule. Motions from the two cases presented by the vape-endorsing entities were denied as the Deeming Rule was found by a federal judge to be within the authority of the FDA [12], which highlights the need for retailers to be aware of the implications of this ruling, i.e., compliance and enforcement periods and deadlines to meet the requirements of the Deeming Rule.

In addition to federal regulations, vape shop retailers (i.e., owners, managers, clerks) must also comply with state-specific regulations (e.g., as of 9 June 2016, California has raised its age requirement for tobacco purchase from 18 to 21 [13] and has also implemented an anti-vape mass media campaign, with the Director of the California Department of Public Health publishing a report on the risks of e-cigarettes [14]) and local tobacco regulations at the city and county level. With all these policies already in or about to take effect, vape shops should be aware of current and impending regulations in order to stay relevant and in business. 

There are some 40 articles on vape shops in the current research literature (e.g., [1,2,3,4,6,15]), but none of those studies have specifically addressed changes in shop policies before and after a change in federal policy regulating e-cigarette sales. The extant research on vape shops examined retailer attitudes and perceptions regarding the safety of vaping, as well as vape shop customer observations [1,2,3,4,6]. A few studies found that most vape shop retailers promoted e-cigarettes as smoking cessation tools [1,6]. Other studies reported that vape shop retailers perceived e-cigarettes to be safer than combustible cigarettes [2,3,6], while one study presented inconclusive results on retailers’ beliefs regarding the health effects of e-cigarettes [4]. 

As of the time when the data were collected, there were no published studies on the extent of brick-and-mortar vape shop retailers’ awareness and beliefs of forthcoming FDA rules and regulations (as well as the extent of retailers’ preparations to meet these requirements). Such studies are important to understand the retailer’s role as a gatekeeper of e-cigarette use and promoters of clients’ switching from combustible to potentially lower-harm products and to help guide support efforts by tobacco control groups focused on improving compliance. To address this gap in the literature, we examined how vape shop store policies and retailer attitudes and beliefs about FDA rules changed over a one-year period from 2014 to 2015. This time period is of particular importance because the FDA announced on 25 April 2014 that it intended to begin regulating e-cigarettes as tobacco products (stated in the form of the proposed Deeming Rule in the Federal Register, a publicly available daily journal of the U.S. government [16]). Popular websites that vape shop retailers and pro-vapers may frequent (e.g., Smoke-Free Alternatives Trade Association (SFATA) [17] and the Vaping Militia) featured FDA activity in blogs and forums, noting imminent FDA regulatory proposals [18,19]. It is feasible that through this and related channels, vape shop retailers would be aware of and thereby prepare to meet these forthcoming regulations. 

### 1.1. Aim 1: Changes over Time

Comparing shops from both time points, we examined changes in: (1) vape shop retailers’ attitudes and beliefs concerning e-cigarettes (i.e., e-cigarette safety and use), (2) vape shop store policies (i.e., offering and/or displaying samples, promotional content, flavor options, employee trainings and nicotine toxicity education), and (3) awareness and support of FDA regulations over a one-year period. Changes in a cautionary direction could indicate increased receptivity to impending regulations. We hypothesized that (1) there would be a negative shift in vape shops’ perceptions of e-cigarette safety over time and (2) increased awareness of FDA regulation. However, although there may be increased awareness of FDA regulations, full compliance (i.e., major change in store operations) may not take effect until the compliance period is in effect. We also hypothesized that (3) there would be only a slight decrease in sample offers/displays but an increase in employee training to prepare for the future potential regulations. 

### 1.2. Aim 2: Year 2 Characteristics

Our second investigation assessed general retailer perceptions, attitudes, and beliefs regarding use of e-cigarettes, as well as awareness of (1) policies created by tobacco-governing entities and (2) pro-vape groups, during the second wave of our data collection. We hypothesized that awareness of federal, state, and local policies would be low (i.e., less than 30%) while awareness of non-political pro-vape groups would be high (i.e., more than 60%), mainly through social media channels. We also hypothesized a difference in awareness of these policies and in general e-cigarette beliefs based on ethnic location.

## 2. Materials and Methods

### 2.1. Shop Recruitment and Data Collection Protocol

Two waves of data collection approved by the Institutional Review Board (IRB) occurred in the Greater Los Angeles area, focusing on predominantly Korean, Hispanic/Latino, African American, and non-Hispanic White communities (identified based on data from the U.S. Census, defined by zip codes and census tracts) to examine vape shop differences as a function of ethnic location [3,6]. The IRB permitted trained research team members to visit sampled vape shops in pairs, asking shop owners or employees available at the time if he or she would like to participate in our study. We assured each employee that the information collected would be kept confidential and would not pose any personal risk, either to the employee or to the shop. We also ensured that conducting the interview would not interfere with the shop’s business by stepping aside during the interview when a customer approached the cashier. After obtaining verbal consent, one research team member conducted the retailer interview, which took about 35 min to complete. One employee was surveyed per vape shop. Simultaneously, the other research team member would record shop characteristics based on an observation checklist (i.e., which items the shop openly displayed, etc.). Those who agreed to participate in our study were compensated with a $50 prepaid gift card.

The first wave of data collection took place from 19 June to 8 December 2014 (Year 1). Prior to data collection, we identified 104 vape shops using reviews from Yelp.com, which excluded shops that also sold combustible tobacco products, such as cigarettes and hookah. Google searches were also used to locate vape shops; however, some results included e-cigarette distributors rather than actual vape shops [3,6,15]. No additional shops were located by Google. Of the 104 initially identified shops, 17 were no longer were in business, four declined participation, four were classified as tobacco shops, one was classified as an e-cigarette distributor, and one was a hookah and vape lounge, resulting in a final count of 77 eligible vape shops. 

After categorizing each vape shop into communities based on racial/ethnic composition, all eligible shops in African American (*n* = 20), Hispanic (*n* = 17), Korean (*n* = 18), and non-Hispanic White (*n* = 22) communities were visited and invited to participate [3,6,15]. Employees at all 77 shops provided consent to participate in our study. 

The second wave of data collection took place from22 July 2015 to 14 January 2016 (Year 2). Of the 77 original shops, 61 remained in-business, and 16 had closed at the start of Year 2 data collection. Employees at all remaining 61 shops provided consent to participate in our study. Year 2 data collection procedures were identical to those of Year 1.

### 2.2. Subjects

The mean age of vape shop employees interviewed was about 27 years of age (SD = 8.34 in Year 1; SD = 9.98 in Year 2), and the majority of them were male in both years (82% in Year 1; 90% in Year 2). Of the employees surveyed during Years 1 and 2, 33% were clerks, 44% were managers, and 23% were shop owners. Of the 17 female employees, 35% were clerks, 53% were managers (higher than 43% male managers), and 12% were owners. Overall, 46% of retailers were Asian, 28% were White, 12% were Hispanic, 11% were Other (e.g., Mixed), and 4% were Middle Eastern.

### 2.3. Measures

#### 2.3.1. Vape Shop Employee-Level Characteristics

During Years 1 and 2 of data collection, participants provided demographic information (e.g., vape shop employee’s age, months of employment, gender, and occupational position, referred to as worker status), personal opinions on e-cigarettes (e.g., “What are your feelings about e-cigarettes in terms of their safety?” (Completely safe; safer than regular cigarettes; about as safe or dangerous as regular cigarettes; or more dangerous than regular cigarettes), “What best describes your feelings about e-cigarettes in terms of their popularity?” (Wave of the future (will keep growing in popularity); current trend (popular now but don’t know future popularity); or temporary thing (popularity will fizzle out in the near-future), “How safe do you think each of the following nicotine-containing products are” on a scale of 1-to-10, with 1 being no danger at all/quite safe to 10 being dangerous/not at all safe?), and self-use information of nicotine-containing products (e.g., ever use, use in the past 30 days, specific types of products, “Did you cut down on/quit cigarette smoking by using e-cigarettes instead?”) The Year 1 survey also included one question on general FDA regulations (e.g., Are you aware of any FDA regulations or other policy statements/actions that might apply to vape shops? Yes or no). 

The Year 2 survey featured questions used during the first wave of collection (FDA regulations in general) and also featured new questions, including employee attitudes about e-cigarettes (i.e., personal right to vape; e-cigarettes are harmful to health; e-cigarettes are harm reduction devices), on a scale of 1 to 10 (with 1 strongly disagreeing, 5 somewhat agreeing, and 10 totally agreeing). Additionally there were questions about whether vape shop retailers favor or oppose proposed e-cigarette regulations (i.e., prohibiting e-cigarette use; using e-cigarette taxes for education; regulating/licensing e-cigarettes as combustible tobacco products are regulated/licensed; restricting e-cigarette flavors), on a scale of 1 to 4 (with 1 strongly favor, 4 opposing strongly, or don’t know/no opinion); believe that both the employee and customers are overly exposed to secondhand vapor; and believe that e-cigarettes and other vaping products contribute/are linked to young people becoming addicted to nicotine.

Regarding policies on e-cigarette sales, we asked if retailers have heard of (1) the Reynolds American’s submission to the FDA that could outlaw rechargeable vape devices [20], (2) the California Senate Bill 140/SB 2X-5, which, if passed, would add to the existing Stop Tobacco Access to Kids Enforcement (STAKE) Act’s definition of tobacco products to include electronic devices, such as e-cigarettes (SB 140/2X-5 was later rejected by the committee) [21], (3) the mass media campaign “Wake Up” by California Department of Public Health (CDPH), which is an advertising movement to counteract e-cigarette use [22], and (4) any local policy statements or actions applying to vape shops; involvement in any activism activities to support the vape shop business; awareness of pro-vape groups and how they find out about these groups; and awareness of any e-cigarette research, either for or against vaping.

#### 2.3.2. Vape Shop Characteristics

We asked about products sold at the shop; the number of flavors and nicotine levels (in mg/mL) sold at the shop; allowance of free trial puffs and samples of nicotine-containing e-cigarettes (and if so, how customers were made aware of these free trials and samples); how e-liquid spills are managed in shops; how shops find out about new e-cigarette products; and deciding factors of which items to keep in the store. 

In order to better understand how e-liquid spills are managed and handled, we asked the following open-ended questions: (1) What type of training do employees receive, and what is taught (e.g., product knowledge, business operations, safety of builds, and nicotine handling)? (2) What are employees told about nicotine toxicity, dangers, and/or treatment if there is a spill? (3) Has there ever been a nicotine-containing e-liquid spill at the shop? (4) Do employees ever touch nicotine-containing e-liquids directly without gloves on? (5) Are you supplied with any safety equipment, such as gloves or goggles? (6) If yes, are they used for contact with nicotine products? [3,6]. In order to simplify results, we dichotomized the first two open-ended questions into yes/no questions (e.g., Do employees receive training? Yes/no. Are employees told about nicotine toxicity if there is a spill? Yes/no).

### 2.4. Analyses

All analyses were performed using SAS 9.4 (SAS Institute Inc., Cary, NC, USA). To assess Aim 1, whether there were differences over time in attitudes and beliefs concerning e-cigarette safety and use and changes in store policies, we conducted simple logistic regression and independent *t*-tests for the categorical and continuous variables of interest, respectively, as well as a Bonferroni correction for multiple tests where relevant. For Aim 2, regarding the opinions and attitudes of vape shop retailers about the FDA asked in Year 2, we assessed the frequencies (proc freq) and means (proc means) of measures. 

## 3. Results

### 3.1. Changes from 2014 to 2015

Awareness of FDA regulations or policies governing e-cigarettes significantly decreased over time (95% in Year 1 to 74% in Year 2; *p* < 0.005; refer to Table 1) among the 61 shops. From Year 1 to Year 2, perceptions of e-cigarette safety significantly shifted from 28% thinking that e-cigarettes were completely safe and 70% thinking they were safer than regular cigarettes to 2% thinking they were completely safe and 98% thinking they were safer than regular cigarettes (*p* < 0.005). All *p*-values remained significant after a Bonferroni correction. Between the two time points, there were no significant differences in perception of e-cigarette popularity, personally cutting down on cigarettes using e-cigarettes, and personally quitting cigarettes using e-cigarettes.

From Years 1 to 2, the number of vape shops displaying free trial puffs through a posted sign/ad significantly decreased (64% to 30%, respectively; *p* < 0.001; refer to Table 2), while the number of shops offering a free trial of e-cigarette puffs directly to the customers face-to-face increased (84% to 98%, respectively; *p* < 0.05). The shops reporting employee training significantly increased between the two time points (84% to 98%, respectively; *p* < 0.05), while reports of nicotine toxicity education remained the same (44% to 42%, respectively; *p* = nonsignificant (NS)). Shops also reported increased incidents of nicotine-containing juice spills at the shop (70% to 97%, respectively; *p* < 0.001) and employees touching nicotine-containing juices directly without gloves on (62% to 89%, respectively; *p* < 0.001). The decrease in displaying a sign/ad for the free trial puff offer; increase in nicotine-containing spills in shop; and increase in employees touching nicotine-containing juices without gloves remained significant after a Bonferroni correction.

Regarding where vape shops get information about new e-cigarette products, there were significant differences from Years 1 to 2 (refer to Table 3). There was a possible increase in relying on social media (82% to 97%; *p* < 0.05), e-cigarette company sales representatives (69% to 85%; *p* < 0.05), and warehouses (46% to 67%; *p* < 0.05) for product information, although these changes were statistically insignificant after applying a Bonferroni correction. From Years 1 to 2, the factors influencing which products to keep in the shop did not significantly differ. Vape shop contact with tobacco company representatives significantly decreased (43% in Year 1 to 23% in Year 2; *p* < 0.05). 

### 3.2. Questions Only Asked in Year 2

All vape shops allowed vaping inside shops, and most of these retailers (83%) did not believe that people could become excessively exposed to nicotine-containing vapor, i.e., secondhand vapor, when inside the shops (refer to Table 4). Most shop employees (73%) reported that they did not have a ventilation system, and if they did, none of the employees knew the brand or model of the system. Only a third (32%) believed that e-cigarettes and other vaping products contribute to young people becoming addicted to nicotine.

When asked about general attitudes toward e-cigarette use, there was a strong agreement among employees (mean = 9.97 out of 10; *p* < 0.001) that one has a personal right to vape. On average, they believed that e-cigarettes are a relatively low danger to one’s health (3.35/10; *p* < 0.001) and strongly agreed that e-cigarettes should be used as harm reduction devices (9.25/10; *p* < 0.001). 

Regarding proposals on the use of e-cigarettes, employees significantly favored prohibiting the use of e-cigarettes and other vaping products in places where smoking is not allowed, such as in restaurants, bars, and workplaces (2.04/4, with 1 = strongly favored, 4 = strongly opposed; *p* < 0.001). They neither strongly favored nor opposed taxing e-cigarettes and other vaping products in California and devoting that money for public education programs, research, and the enforcement of laws relating to their use (2.51/4; *p* = NS). Employees were slightly more opposed to a proposal to regulate and license shops that sell e-cigarettes and other vaping products in California in the same way as stores that sell regular tobacco cigarettes (2.73/4; *p* = NS). On average, they strongly opposed passing a state law that restricts adding flavors to e-cigarettes and other vaping products to reduce their appeal to young people (3.85/4; *p* < 0.001).

Concerning vape-related proposed legislation other than the Deeming Rule, nearly half (48%) had heard of the California Senate Bill 140. Nearly two thirds (62%) were familiar with the California Department of Public Health’s mass media campaign “Wake Up”. Almost 40% of employees had heard about other local policy statements or actions that might apply to vape shops, and 45% had been involved in activism activities to support vape shop business. Besides policy, 47% were aware of e-cigarette research, either for or against vaping.

Regarding knowledge of pro-vape groups for Aim 2, more than half of vape shop employees (59% and 57%, respectively) had heard of Consumer Advocates for Smoke-free Alternatives Associations (CASAA) and Smoke Free Alternatives Trade Association (SFATA), two of the largest pro-vape advocacy organizations [18,23]. However, only 36% had heard of Vaping Militia, 23% of Vapefreeyouth.com, and 28% of any other groups, which included IM PROOF, Cloud Kicker Society, Notblowingsmoke.org, United Vapers, and American E-Liquid Manufacturing Standards Association (AEMSA). Employees found out about these groups mostly through word of mouth (57%) and Instagram (49%), with a few finding out through Facebook (34%), forums (25%), and other sources (21%), such as Reddit, conventions, YouTube, and other vaping events. 

Overall, there were no significant differences in vape shop employee awareness, perceptions, and attitude toward FDA-related tobacco policies; awareness of other vape-related policies; and general beliefs on e-cigarette across ethnic communities.

## 4. Discussion

Our analyses show that vape shop employees (1) generally believe that e-cigarettes are relatively safe to use and (2) have a good understanding of local regulations but still have gaps in their understanding of impending federal regulations.

### 4.1. Changes (Individual- and Shop-Level) over Time

In terms of e-cigarette safety perceptions, one held by the vast majority (98%) of the employees by the second time point, that e-cigarettes are not completely safe but rather safer than combustible cigarettes, has implications for their interactions with customers when explaining the safety of using e-cigarettes and related vape products. On a related note, fewer vape shop employees reported personally cutting down on or quitting combustible cigarette smoking in the second wave compared to the first, which may be related to the safety perceptions. 

The significant increase in employees directly telling customers of free e-cigarette puff trial offers rather than posting displays of the offers in the shop suggest retailers are finding ways around the impending FDA regulations by not having displays but still engaging in the (soon-to-be illegal) behavior. This may signify that awareness of, but not necessarily compliance with, the impending rules is occurring in the vape marketplace; therefore, FDA may need to focus on marketplace outreach efforts aimed to increase compliance since awareness is present. Although not significant, the increasing trend among shops in selling premixed juices may also support this increase in awareness of rules and regulations.

Although the majority of shops provided general employee training and acknowledged the high importance of employee safety, less than half of the shops trained their employees on nicotine toxicity, dangers, or treatment if there was a spill. A recent study highlighted that vape shops’ e-liquid handling practices posed a potential occupational hazard [19]. Some vape shop employees believed nicotine to not be toxic at all or that only the high nicotine doses were dangerous, according to participants’ open-ended interview answers. This is highly concerning in terms of employee and customer safety during spill incidents. With elevated rates of nicotine-containing e-liquid spills in shops and not all shops providing safety equipment (e.g., gloves, goggles)—and some employees even touching the nicotine-containing spills without having gloves on—the FDA could consider providing a safety training component to raise awareness of the dangers and toxicity of nicotine at any dose and of the occupational hazards at a vape shop, as well as either providing the actual safety equipment needed or directing retailers to the appropriate safety equipment providers. 

### 4.2. Awareness of Federal/State/Local Rules and Information Sources to Find Out about These Policies

Contrary to our hypothesis that there would be increased awareness of FDA regulations, awareness of impending FDA regulations actually significantly decreased over time among vape shop employees. Based on the observation that all shops continued to offer free trial e-cigarette puffs even though prohibited by the impending regulations, this supports our other hypothesis that there would not necessarily be full compliance (i.e., shops may wait and keep current store operations for as long as possible until the compliance period goes into effect and is actually enforced). The results suggest that the FDA needs to raise awareness and communication efforts concerning the Deeming Rule and other policy changes with vape shops. 

Contrary to our hypothesis that there would be relatively low awareness of policies created by state/local tobacco-governing entities but high awareness of pro-vape groups, overall awareness of state/local policies was higher than expected (average awareness of 45%, ranging from 31% to 62%), while overall awareness of pro-vape groups was lower than expected (average awareness of 41%, ranging from 23% to 59%). 

As we hypothesized vape shops’ main source of information on e-cigarette products being social media channels, there was a possible increase in relying not only on social media but also on e-cigarette company sales representatives and vape product warehouses, which may have played a key role in informing vape shops of new e-cigarette products and in guiding shops on which items to offer and sell. The FDA could consider reaching out to both in-store and online vape shops through social media channels and outlets in the future, such as Twitter (i.e., popular and/or trending hashtags associated with vape shop activities or e-cigarettes) or Facebook, and partner with e-cigarette companies and warehouses/distributors to ensure consistency of information and increase compliance with pending and current rules and regulations. 

### 4.3. Community Differences in Awareness and Study Limitations

Furthermore, although we hypothesized a difference in awareness of vape-related policies and general e-cigarette beliefs based on ethnic location, the data showed non-significant results. This may have been due to a small sample size and not enough power to detect ethnic location differences. Future studies should sample larger numbers of vape shops from different regions of the U.S. and elsewhere to increase external validity of the data and to detect statistically significant trends in retailer practices and beliefs.

Another limitation of this study was the inability to identify whether or not the employee surveyed in Year 2 was the same employee surveyed from Year 1. In order to protect the employee’s privacy and anonymity, we could not document his or her identity (i.e., name) during data collection. Although we were originally interested in focusing on the individual, we had to follow the shop rather than the individual due to this data limitation. The individuals were selected because they work for the shop, so their beliefs and awareness may at least somewhat reflect views of that worksite. While these results describe changes over time in vape shops in the Greater Los Angeles area, the results may be harder to apply and/or less relevant to other areas, especially given the diverse regulatory environments in different countries with varying degrees of e-cigarette usage. 

Since the completion of the current study, several state-level policies were enacted (STAKE Act and CPC Section 308; SB5 X2, SB7 X2, and AB11 X2) and took effect in 2016: vape shops (a) are not allowed to sell e-cigarette devices or liquids to persons under 21 years of age (and need to check photo IDs), (b) they must post an age-of-sale warning sign by cash registers, (c) it is illegal to have a self-service display in the shops for e-cigarette products, (d) all e-device cartridges and juices used for filling devices must be sold in child-resistant packaging, and (e) shops need to apply for a state tobacco retail license (i.e., if they qualify as a retail/wholesale tobacco shop, or private smokers’ lounge, then vaping is allowed in the shop). Thus, our findings may have included reactions to both impending state as well as FDA actions. A subsequent wave of post-policy data collection is warranted to better address awareness and compliance with recent federal and state policies.

## 5. Conclusions

This is the first longitudinal study of vape shop retailers to measure differences in vape shop attitudes, beliefs, and actions related to anticipation of the FDA’s Deeming Rule. We found that retailers generally believe that e-cigarettes are relatively safe to use, which is crucial considering that retailers are the gatekeepers to new or current vaping behavior. Although there may be higher overall awareness of local policies, most retailers were not quite as aware of federal regulations. 

Exploring whether there has been changes in store policies and retailer attitudes/beliefs over time can provide guidance to tobacco control organizations to guide efforts to educate retailers on current and future regulations and provide support that will improve compliance. Future research should continue to survey and observe the ever-changing tobacco marketplace landscape and incorporate questions to assess changes in vape shop operations and compliance with the Deeming Rule. It would be important to examine if vape shops are complying with the imminent regulations that will be effective starting 10 May 2018: vape shop retailers will be prohibited from selling/distributing e-cigarettes or other vape products without including a health warning statement on the product packaging and from displaying e-cigarette or other vape product advertisements without including a health warning statement on the ads [11].

## Figures and Tables

**Table 1:** Surveyed vape shop employees’ demographics, perceptions of e-cigarettes, personal vaping behaviors, and awareness of FDA regulations (*n* = 61) using *t*-test or simple logistic regression.

**Variable**	**Subvariable**	**Year 1 (*n* = 61) (Mean (SD); Range)**	**Year 2 (*n* = 61) (Mean (SD); Range)**	**Significance (*p*-Value)**
Age (in years) ^a^		27.57 (8.34); 18–59	28.15 (9.98); 17–72	0.731
Months of employment ^a^		11.29 (5.00); 1–24	19.39 (10.19); 1–36	<0.001 *^,B^
Safety perceptions of nicotine-containing products, on a scale from 1 to 10 (1 signifying no danger at all/quite safe; 10 dangerous/not safe) ^a^	Cigarettes without filters	9.54 (1.07); 5–10	9.46 (1.21); 5–10	0.692
	Cigarettes with filters	9.00 (1.26); 5–10	8.70 (1.77); 2–10	0.292
	E-cigarettes (disposables)	3.77 (2.43); 1–10 ^c^	4.54 (2.31); 1–10	0.075
	E-cigarettes (rechargeables)	2.66 (1.76); 1–8	2.67 (1.34); 1–5	0.954
**Variable**	**Subvariable**	**Year 1 (*n* = 61) (Frequency (%))**	**Year 2 (*n* = 61) (Frequency (%))**	**Significance (*p*-Value)**
Gender ^b^	Male	50 (82)	55 (90)	0.197
	Female	11 (18)	6 (10)	
Worker status ^b^	Owner or Manager	37 (61)	42 (69)	0.344
	Clerk	24 (39)	19 (31)	
Perception of e-cigarette safety ^b^	Completely safe	17 (28) ^c^	1 (2)	0.002 *^,B^
	Safer than regular cigs	43 (72)	60 (98)	
Perception of e-cigarette	Wave of the future	54 (90) ^c^	53 (87)	0.811
popularity ^b^	Current trend	6 (10)	8 (13)	
Personally cut down on cig smoking using e-cigarette ^b^	Yes	55 (90)	54 (89)	0.769
Personally quit cig smoking using e-cigs ^b^	Yes	52 (85)	45 (74)	0.121
Aware of new FDA regulation ^b^	Yes	58 (95)	45 (74)	0.004 *^,B^

^a^ Independent *t*-test to test significance of difference between the two years. ^b^ Simple logistic regression to test significance of difference between the two years. ^c^ One missing response omitted (*n* = 60). * Significant *p*-value. ^B^ *p*-value remains significant after a conservative Bonferroni correction for multiple tests (0.005).

**Table 2:** Vape shop characteristics (*n* = 61) using *t*-test or simple logistic regression.

Variable	Subvariable	Year 1 (*n* = 61) Mean (SD); Range or Frequency (%)	Year 2 (*n* = 61) Mean (SD); Range or Frequency (%)	Significance (*p*-Value)
Number of e-cig flavors ^a^		127.60 (77.20); 25–400	114.60 (66.40); 20–300	0.321
Nicotine levels sold (mg/mL) ^b^	0	59 (98) ^c^	61 (100)	0.959
	3	46 (77) ^c^	61 (100)	0.934
	6	57 (95) ^c^	61 (100)	0.953
	12	58 (97) ^c^	60 (98)	0.557
	18	51 (85) _c_	45 (74)	0.131
	20	3 (5) ^c^	3 (5)	0.983
	24	25 (42) ^c^	16 (26)	0.075
Free trial e-cig puffs ^b^	Yes	61 (100)	61 (100)	N/A
Method of offering	Display	39 (64)	18 (30)	<0.001 *^,B^
free trial e-cigarette puffs ^b^	Face-to-face	51 (84)	60 (98)	0.021 *
Free nicotine e-cigarette sample ^b^	Yes	34 (56)	30 (49)	0.469
Sell dry herb atomizers, vaporizers, or pens ^b^	Yes	19 (31)	19 (31)	N/A
Customers mix e-juice ^b^	Yes	14 (23)	22 (36)	0.115
Shop mixes e-juice ^b^	Yes	17 (28)	14 (23)	0.533
Sell pre-mixed e-juice ^b^	Yes	55 (90)	60 (98)	0.087
Employee trainings ^b^	Yes	51 (84)	59 (98) ^c^	0.022 *
Nicotine toxicity education ^b^	Yes	27 (44)	25 (42) ^c^	0.773
Nicotine spills in shop ^b^	Yes	43 (70)	59 (97) ^c^	0.001 *^,B^
Touch nicotine in shop	Yes	38 (62)	54 (89)	0.001 *^,B^
Safety equipment (SE)	Yes	51 (84)	46 (75)	0.265
SE for nicotine contact	Yes	28 (55) ^d,e^	27 (59) ^e^	0.369

^a^ Independent *t*-test. ^b^ Simple logistic regression. ^c^ One missing response omitted (*n* = 60). ^d^ Eleven missing responses not omitted for count (i.e., 28 out of 61 answered yes). ^e^ Percentages out of those who answered yes: Year 1: 28/51 = 55; Year 2: 27/46 = 59. * Significant *p*-value. ^B^ *p*-Value remains significant after a conservative Bonferroni correction for multiple tests (0.006).

**Table 3:** Informational sources and deciding factors influencing marketed vape shop items (*n* = 61) using simple logistic regression.

Variable	Subvariable	Year 1 (*n* = 61) Frequency (%)	Year 2 (*n* = 61) Frequency (%)	*p*-Value
Info sources about new e-cigarette products	Internet	51 (84)	57 (93)	0.099
	Social media	50 (82)	59 (97)	0.018 *
	Personal contacts	48 (79)	53 (87)	0.234
	Sales reps	42 (69)	52 (85)	0.035 *
	Warehouses	28 (46)	41 (67)	0.019 *
	Other ^a^	7 (11)	8 (13)	0.783
Deciding factors on which products to keep in shop	Internet	26 (43)	20 (33)	0.263
	Social media	32 (52)	33 (54)	0.856
	Personal contacts	27 (44)	19 (31)	0.137
	Sales reps	18 (30)	15 (25)	0.541
	Warehouses	11 (18)	13 (21)	0.649
	Other ^b^	14 (23)	21 (34)	0.163
	Based on sales	49 (80)	56 (92)	0.075
Tobacco company representative ^c^	Yes	26 (43)	14 (23)	0.022 *

^a^ Other: Magazines, personal preference, trade shops, corporate relationships with manufacturers, wholesalers, customers, distributors, e-juice companies, YouTube, expos and conventions, events, travelling to countries offering new products. ^b^ Other: Device/juice safety, inventory, monthly shop meetings, self-testing, customer feedback, owner preference, trial and error, distributors, ease of device use, based on demographics, word of mouth, conventions, trusted companies. ^c^ Have you had any contact with tobacco industry agents, such as distributors of cigarette and/or e-cigarette products? * Significant *p*-value. Note: There were no *p*-values remaining significant after a conservative Bonferroni correction for multiple tests (0.008 for info sources; 0.007 for deciding factors; N/A for tobacco company representative).

**Table 4:** Vape shop employees (*n* = 61) from Year 2 only: general attitudes and beliefs of e-cigarettes and policies; awareness of e-cigarette/pro-vape groups; information sources of these groups; and other related items.

**Variable**	Mean (SD); *p*-Value *
General attitudes ^a^ (*n* = 60)	
Personal right to vape	9.97 (0.18); <0.001
E-cigs harmful to health	3.35 (2.25); <0.001
E-cigs as harm reduction	9.25 (1.40); <0.001
Beliefs on e-cig policies ^b^ (*n* = 55)	
Prohibiting e-cig use	2.04 (1.02); <0.001
Taxing for education	2.51 (0.90); 0.931
Regulating/licensing	2.73 (1.27); 0.162
Restricting flavors	3.85 (0.49); <0.001
**Variable**	**Yes (%)**
General beliefs (*n* = 61)	
Secondhand vape ^c^	10 (16.67)
E-cigarette and nicotine addiction link ^d^	19 (32.20)
Awareness of e-cig policies (*n* = 61)	
Reynolds American policy	19 (31.15)
CA SB 140	29 (47.54)
CDPH “Wake Up”	38 (62.30)
Local action	24 (39.34)
Awareness of Pro-vape groups (*n* = 61)	
CASAA	36 (59.02)
SFATA	35 (57.38)
Vaping Militia	22 (36.07)
Vapefreeyouth.com	14 (22.95)
Other	17 (27.87)
Info sources (*n* = 61)	
Instagram	30 (49.18)
Facebook	21 (34.43)
Forums	15 (24.59)
Word of mouth	35 (57.38)
Other	13 (21.31)
Other e-cig-related items (*n* = 61)	
Activism ^c^	27 (45.00)
E-cig research ^c^	28 (46.67)
Ventilation in shop ^d^	16 (26.23)

* One-sample *t*-test to see if mean is different from midpoints (5.5 for general attitudes and 2.5 for beliefs on policies). ^a^ Scale for “General attitudes”: 1: Strongly disagree, 5: Somewhat agree, 10: Totally agree. ^b^ Scale for “Favoring/opposing”: 1: Favor strongly, 2: Favor somewhat, 3: Oppose somewhat, 4: Oppose strongly. ^c^ Missing values: 1 (*n* = 61). ^d^ Missing values: 2 (*n* = 61). CDPH: California Department of Public Health; CASAA: Consumer Advocates for Smoke-free Alternatives Associations; SFATA: Smoke-Free Alternatives Trade Association.
